# Evaluating GP trainees’ perceptions of teaching undergraduate medical students on clinical placements: a thematic analysis

**DOI:** 10.1186/s12909-026-09820-5

**Published:** 2026-07-01

**Authors:** Sarah Merrifield, Eleanor Lister, Sophia Chaudhry, Samuel Mottaghi-Taromsari, Tal Wasty

**Affiliations:** 1 MBChB Community Team, University of Manchester, Stopford Building, Manchester, M13 9PT England; 2 Royal Bolton Hospital, Minerva Road, Bolton, BL3 0JR UK; 3 Firdale Medical Centre, Firdale Road, Northwich, Cheshire, CW8 4AZ England

**Keywords:** Near-peer teaching, Undergraduate medical education, General practice, Clinical placement supervision

## Abstract

**Background:**

There is a recognised desire to teach amongst GP trainees yet limited formal opportunities to do this. There are known benefits of near-peer teaching to both teacher and learner, but minimal research investigating the role of GP trainees as teachers in a clinical setting. This study aims to evaluate the views of GP trainees participating in a teaching scheme piloted at the University of Manchester.

**Methods:**

This mixed-methods study was conducted over a three-year period. In years one and three, evaluation was undertaken using questionnaires comprising Likert scale items and free-text responses (*n* = 11). In the second year, qualitative data was collected via a focus group with GP trainees following their teaching experiences (*n* = 8). Subsequently, a thematic analysis was conducted.

**Results:**

GP trainees reported benefits including improved clinical skills, job satisfaction and development of leadership skills. There was a noted impact on their teaching skills, as GP trainees demonstrated their ability to be adaptable to learners’ needs and develop interactive, structured teaching sessions.

The main challenges identified were organisation and time management, balancing responsibilities and adapting to student needs. Trainees unanimously felt the scheme allowed them to meet their portfolio requirements, develop their teaching skills and made them more likely to consider pursuing medical education as part of their future careers.

All participants expressed a desire to continue with teaching and reported improved job satisfaction.

**Conclusion:**

Participation in a near-peer teaching scheme can have significant positive impacts on GP trainees’ personal and professional development. Despite some challenges, trainees reported numerous benefits from undertaking the scheme. Formal teaching opportunities for GP trainees could contribute to the development of a skilled and motivated future workforce of clinician-educators in general practice.

**Supplementary Information:**

The online version contains supplementary material available at https://doi.org/10.1186/s12909-026-09820-5.

## Background

A GP trainee is a qualified doctor who is undertaking vocational training in general practice (GP). They can also be referred to as a GP Specialist Trainee (GPST), or GP registrar. Current full-time training in the United Kingdom usually lasts three years during which the trainee is expected to meet specified curriculum requirements and undertake a range of assessments. On satisfactory completion, the doctor attains a Certification of Completion of Training (CCT) to enter onto the GP register and Membership of the Royal College of General Practitioners (MRCGP). Supporting the education and professional development of colleagues is one of the specific capabilities for general practice listed in the Royal College of General Practitioners (RCGP) curriculum [1]. Undergraduate medical students spend a significant portion of clinical time in general practice as a fundamental part of their clinical learning experience. The increasing capacity of medical students and the demand for good quality placements has placed substantial strain on an already overburdened system [2]. GP registrars have been identified as an underutilised resource [3].

There is an evident desire for GP registrars to engage with teaching in general practice [3, 4]. Studies have identified a significant proportion, ranging from 50% to 80% of GP registrars reporting a desire to teach [5–7]. GP trainees have been noted to have limited opportunities to teach in relation to their hospital-based peers [8].

Whilst there are a few formal GP trainee led teaching schemes [9, 10], most teaching opportunities are self-organised and do not include targeted training, quality assurance or debrief. There is minimal research investigating the role of GP trainees as teachers in a clinical setting which is arguably the area in greatest need of quality medical educators.

There is substantial evidence of the benefits of near peer teaching [11–13]. Weiss showed greater knowledge acquisition from tutors compared to tutees following educational sessions, evidencing the adage “to teach is to learn twice” [11]. Through teaching, there is the potential for GP registrars to recognise their individual learning styles and address their academic needs [4, 5] in addition to developing teaching skills.

There is some suggestion that GP trainees lack confidence to teach. Previous findings have indicated this can be attributed to their trainer’s opinion and lack of training [6, 14]. Another key barrier echoed by GP trainees was their ability to balance teaching with their own learning needs. GP trainees are placed under pressure to balance their responsibilities as a clinician and their needs as learners in their own right [5, 15]. Many GP registrars have reported a lack of time to partake in teaching. Those who can commit to teaching may struggle to continue their responsibilities as near peer teachers when exams near [14, 16].

Since 2022, the University of Manchester in the United Kingdom has run a GP registrar led teaching pilot called GPST Education and Mentoring (GPSTEAM). The pilot was introduced both to provide GP trainees with structured opportunities to develop their teaching skills and to increase teaching capacity for Year 1 undergraduate placements, while also enhancing the student experience through a near-peer teaching model supported by existing educational evidence. GP registrars in their final year of training from local training programmes were offered this as a voluntary opportunity with support of their trainer. Participating GP registrars were provided with training to provide placement supervision. This was a 3-hour session that included the University’s Professionals in Medical Education (PRiME) training for new supervisors. Content included giving effective feedback, supervision on clinical placement, an introduction to clinical reasoning and information on the wider MBChB program and student expectations. Completion of this training allows participants to undertake supervision post CCT.

Participants facilitated pairs of Year 1 medical students undertaking early clinical experience (ECE) full day visits in general practice. ECE visits showcase the roles of a General Practitioner and how a GP surgery runs, with student opportunities for patient encounters and clinical skills development. Participants were offered flexibility in the number of visits to meet their work schedule and educational needs. Between 2022 and 2025, 27 GPSTs undertook the scheme, supervising approximately 100 Year 1 students on placement. The number of GPST participants varied between 6 and 14 annually. This project set out to explore the perceived benefits and challenges of trainees as near-peer medical educators in a clinical supervision setting.

## Methods

An interpretivist approach was adopted to study GP trainees’ subjective experiences of teaching, with the study’s primary objective to collate the perceived benefits and challenges of GP trainees as near-peer medical educators. A qualitatively driven mixed-methods design was used. Qualitative data through focus group work was the primary methodological approach, with GP trainee questionnaire data to add to these findings. The focus group enabled GP trainees to discuss and reflect on their experiences collectively, allowing exploration of both shared and divergent perspectives regarding their experiences as undergraduate educators.

GP trainees were recruited for the scheme via an email sent out to the GP School North West Deanery whereby those interested completed a form to express their interest and, if permission was granted from their training practice and GP trainer, they were then recruited to a training day to prepare for the supervisor role. GPSTs participated in the scheme each year over a 3-year period from 2022 to 2025. The number of visits they each supervised ranged between 1 and 8. In total 11 tutors from the 2022–2023 and 2024–2025 cohorts completed an online questionnaire measuring their confidence in key skills relating to both general practice and medical education after their experience of GPSTEAM. This questionnaire was developed specifically for this project involving both Likert scale and free text responses (see supplementary file “Questionnaire”).

The evaluation questionnaire was designed principally to answer the core question of this study, to explore the benefits and challenges for trainees as near-peer medical educators in a clinical setting. Therefore, the style of questions adopted in the written questionnaire was primarily focussed on Likert scale responses (and fewer free-text questions) to allow for a quantitative overview, complemented later by focus groups for more in-depth, rich, qualitative data analysis.

Questions were written in adherence with basic evaluation design principles to improve reliability, such as alignment to objectives, use of clear and simple language, avoidance of leading and multi-factorial questions, and use of appropriate and balanced response scales. Questionnaire length and structure were also considered to avoid responder fatigue without significantly compromising the validity of data being collected.

Attempt was made to evaluate longer term impact by including questions that would gauge aspirational behaviour change and aspirational results as a consequence of those behaviour changes.

Eight participants (of a cohort of 13) from the 2023–24 academic year of the scheme voluntarily attended an in-person focus group at the University of Manchester after an invitation was sent by e-mail. A participant information sheet was provided prior to the event by e-mail and all participants provided written consent. The focus group was facilitated by one of the academic leads for the scheme. Speakers were numbered 1–9 with speaker 1 being the academic lead. The feedback was recorded on a university dictaphone. The data was transcribed, anonymised and stored on a secure university drive.

The transcript was then thematically analysed using Braun and Clarke’s six-phase thematic analysis framework, including familiarisation with data, generating initial codes, searching for themes, reviewing themes, defining and naming themes, and producing the report [17]. The two researchers who undertook the thematic analysis were General Practitioners and medical educators. The thematic analysis approach is consistent with an interpretivist orientation, acknowledging the themes were actively generated through researchers’ engagement with the data, and ongoing reflexive discussions were undertaken throughout the analytical process.

The following questions were used as a framework for discussion:

1. What have been the benefits of teaching undergraduate students?
2. What have been the challenges of teaching undergraduate students?
3. What has this teaching opportunity taught you about yourself?
4. Has this teaching opportunity impacted on your professional goals or development? If so, how?
5. Has this teaching opportunity impacted on your personal goals or development? If so, how?
6. Overall, how have you found your teaching experience with ECE?

## Results

Eleven tutors from the 2022–2023 and 2024–2025 cohorts completed the online questionnaire. Their average scores for perceived development in key skills are outlined in the table below (Table 1). Trainees responded to the question on a 5-point likert scale (1 = Strongly disagree, 5 = Strongly agree).

**Table 1:** GP trainees’ perceptions on skills development following their GPSTEAM experience. The average score out of 5 noted across the 11 responses to each question by the GP trainees

I felt being the main supervisor for students helped me:	GPST Average Score (/5)
Develop My Own Clinical Learning	4.36
Fulfil My Own GP Training Portfolio Requirements	4.64
Develop My Teaching Skills	4.64
Develop Role Modelling and Leadership Skills	4.64
Develop Time Management Skills	4.45
Develop Organisational Skills	4.55
Develop Communication Skills	4.55

**Fig. 1:**
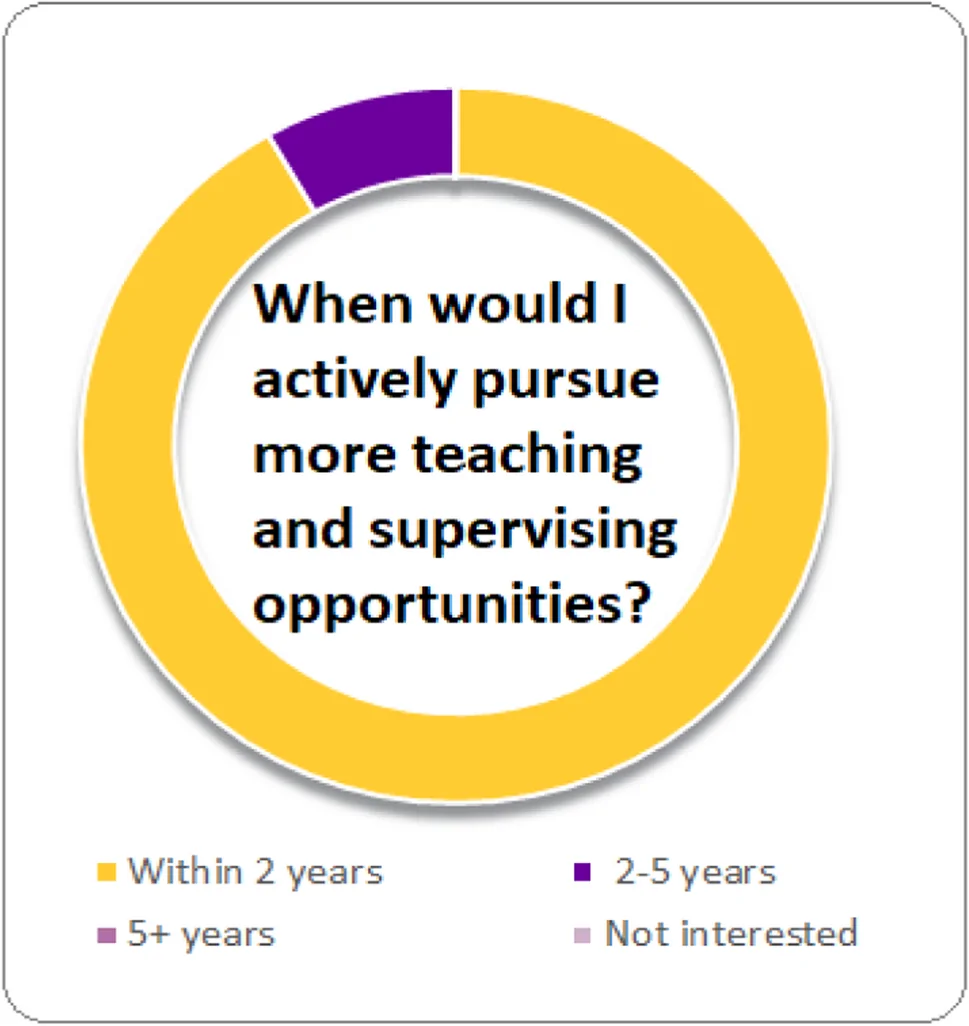
GP trainee perceived timeline on when to pursue teaching and medical education roles

Within the questionnaire GP registrars were also asked to state how soon they would want to pursue more teaching and medical education opportunities. 91% of respondents wanted to pursue these opportunities within 2 years and the remaining 9% wanted to pursue in 2–5 years, as shown in the chart below (Fig. 1).

**Fig. 2:**
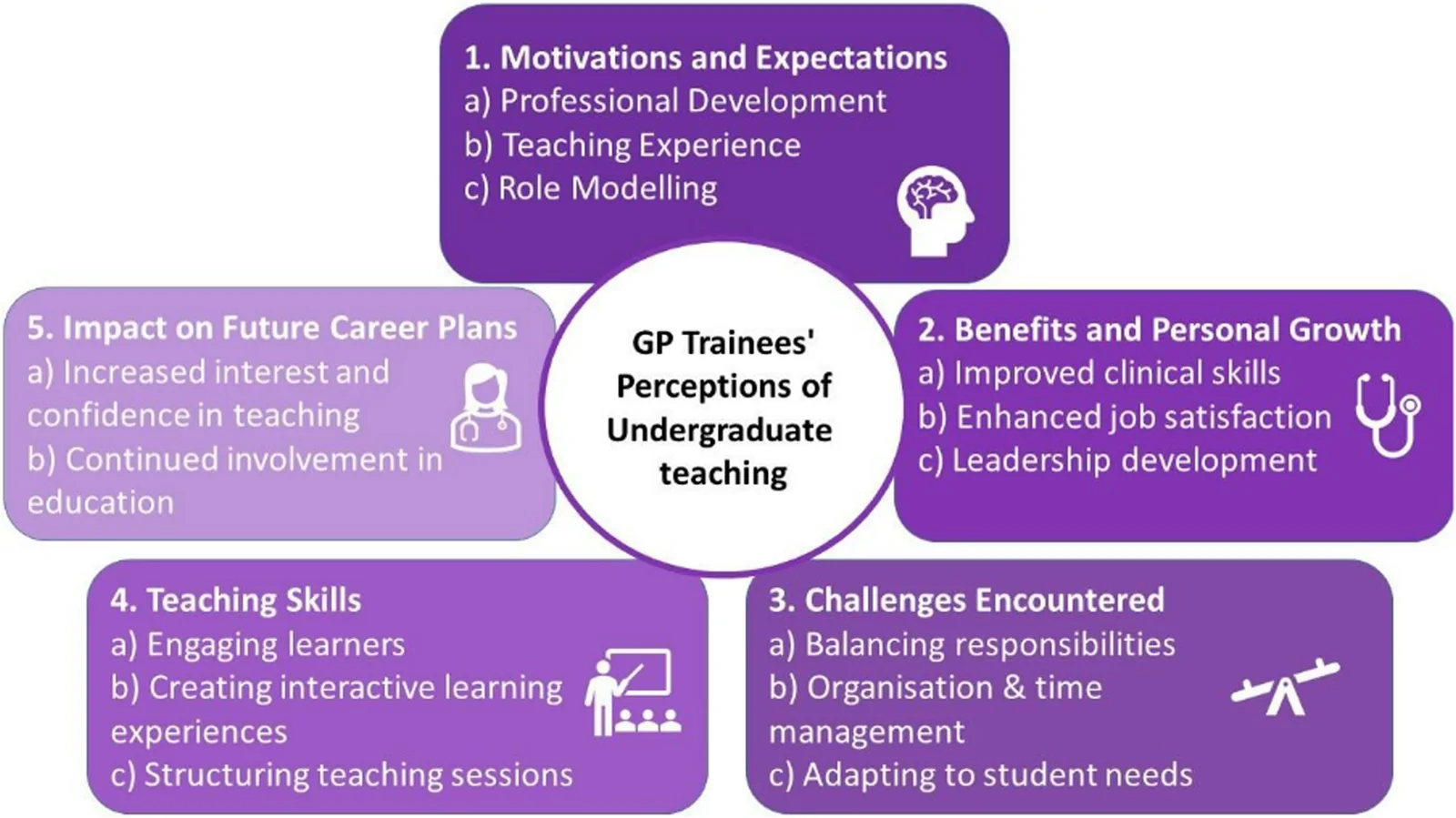
Themes and subthemes identified from analysis of the transcript

Following thematic analysis of the focus group, the broad themes and subthemes are shown below (Fig. 2).

### Theme 1: motivations and expectations

Participants described motivations centred around professional development, gaining teaching experience, and acting as role models for students.

Many trainees viewed the scheme as an opportunity for professional growth. Speaker 2 explained: “I think I just wanted more of an exposure in terms of what supervising is like, and then also ticking the box for my leadership project”.

Participants also described a desire to promote general practice and provide positive role modelling for medical students. Speaker 7 stated: “I want to role model it… But I also want to improve the image of GPs in front of, well, in front of society in general.”

Some trainees expressed concerns about their suitability and ability to teach. Speaker 9 reflected:

“…will I be suitable for teaching students in the UK and Manchester, you know? Would I be too old? Will they get bored? Would they completely hate general practice going away?”

### Theme 2: benefits and personal growth

Participants reported benefits including enhanced clinical practice, increased job satisfaction, improved wellbeing, and leadership development. Several trainees felt teaching improved their clinical practice. Speaker 8 commented: “I think it makes you a better clinician actually… you want to do a good job to show them what it’s like”.

Similarly, Speaker 2 described how teaching reinforced their own knowledge and confidence: “You’re solidifying your knowledge because you can’t make an answer up. And also you do have to, like, back your answer up…”.

Teaching was also perceived as enhancing job satisfaction and wellbeing. Speaker 9 explained: “I think it probably helps you feel a bit more good. It takes you back to when you first wanted to be a doctor, before all of the mess and everything.” Speaker 8 added: “Because they make you feel appreciated. Which you often don’t get I think.”

Leadership development was another frequently reported benefit. Speaker 7 noted: “every step helps you step into that leadership role …I found that quite - very - helpful.”

This was supported by questionnaire data, where most participants strongly agreed that the scheme had enhanced their leadership and role-modelling skills.

### Theme 3: challenges encountered

Although challenges were infrequently reported, participants described difficulties balancing clinical and teaching responsibilities, organisational demands, and adapting teaching to different learner needs.

Speaker 8 reflected: “For me it was that before my sole focus was teaching, whereas now you have to balance the clinical and making sure that you’re doing the right thing for the patient as well as managing two students.”

Organisational challenges were highlighted by Speaker 3: “For me it was organisation. So I tried to do it like a rotation system where they’d have time with the admin staff, time with the nurses, and then me at the end.”

Participants also discussed adapting teaching to learners with different backgrounds and prior experience. Speaker 8 stated: “I was going in at a very basic level and then when I asked them about their previous experiences…”.

The same participant emphasised the importance of: “knowing your learner, being adaptable, pitching at the right level for them”.

### Theme 4: teaching skills acquired

Participants described developing a range of educational skills, including creating interactive learning experiences, adapting teaching approaches, and structuring learning opportunities.

Speaker 2 explained: “constantly trying to keep it interactive and keeping their attention because I think like you guys said before, I was really bored in my medical student placement…”.

Another example of active teaching strategies was described: “We did practice OSCE stations that I just randomly made up at the time between our breaks. It’s really good fun.”

Questionnaire data also demonstrated high levels of agreement regarding teaching skills development. One respondent commented: “I learnt a lot from it and doing this will set me up for future teaching opportunities.”

### Theme 5: impact on future career plans

Many participants reported increased confidence in pursuing future educational roles.

Speaker 9 stated: “I think I would always want to do medical education, but I didn’t know how to do it or if I would be any good at it so then this gave me more confidence to do the things that I want to do.”

This was reflected in questionnaire findings, with 91% of respondents reporting plans to pursue further teaching or supervision opportunities within the next two years.

Participants also linked teaching to future career satisfaction. Speaker 3 reflected: “Yeah, 100% really been enjoyable. I definitely want to continue this in the future…”.

## Discussion

The findings reveal five key themes that highlight the impact of near-peer teaching on GP trainees’ professional development, wellbeing, educational skills, and future career aspirations. Overall, participants perceived the scheme as a valuable opportunity that enhanced confidence in teaching, promoted leadership development, and increased enthusiasm for future educational involvement.

One strength of this study is its mixed-methods design, which enabled triangulation of focus group data, questionnaire responses, and free-text comments, providing a richer understanding of trainees’ experiences. Reflexive consideration during thematic analysis further strengthened the trustworthiness of the findings.

The study provides evidence predominantly at Level 1 (reaction) of Kirkpatrick’s evaluation model [18], with participants reporting highly positive perceptions of the teaching experience. There were also indications of Level 2 (learning), including perceived development of leadership, supervision, and teaching skills. However, the study was not designed to evaluate behavioural change (Level 3) or downstream educational outcomes (Level 4). Future research should explore whether participation as a near-peer educator influences subsequent teaching behaviour, educational career choices, teaching competence, and student learning outcomes.

The GP trainees’ motivations for engaging in the scheme were largely centred around professional development and role modelling. These findings align with previous literature highlighting the reciprocal benefits of near-peer teaching for both learners and teachers, including leadership development and enhanced understanding of subject matter [19]. The desire to act as positive ambassadors for general practice is particularly noteworthy given concerns regarding recruitment into the specialty and the influence of role models on career choice [20]. Role-modelling may also facilitate students’ professional identity formation [21]. Through mentorship and participation in clinical activities, students are exposed to the values and behaviours of the profession, which can shape their developing professional sense of self. It is, however, worth noting, that some trainees expressed uncertainty about their suitability to teach, reflecting previous reports that GP trainees may lack confidence in their educational and mentoring abilities [6, 14].

Participants described a range of personal and professional benefits, including enhanced clinical practice, increased job satisfaction, improved wellbeing, and leadership development. These findings support previous evidence suggesting that teaching and supervision roles can reinforce clinical knowledge and contribute positively to professional development [22]. The reported improvements in job satisfaction are particularly relevant given ongoing concerns regarding burnout and workforce retention within primary care [23, 24]. Similar benefits have been described both in trainee-led teaching initiatives [10] and in clinical supervision roles more broadly [25].

Leadership development emerged as a prominent finding. Participants reported increased confidence in leadership and role-modelling responsibilities, supporting previous evidence that educational roles can facilitate leadership skill development among healthcare professionals [26]. Given the recognised importance of clinical leadership within primary care, these findings suggest that near-peer teaching schemes may provide valuable opportunities for developing future leaders within the profession [19].

Although challenges were relatively limited, participants described difficulties balancing teaching and clinical responsibilities, alongside the need to adapt teaching to learners with differing levels of experience. These findings mirror challenges reported by clinical supervisors more broadly [27] and suggest that supported educational roles during training may help develop adaptability, organisation, and supervisory skills before trainees assume more formal educational responsibilities.

The teaching approaches described by participants reflected principles associated with effective near-peer education, including adaptability, active learning, and educational congruence. These findings support existing literature suggesting that near-peer educators may be particularly well positioned to create engaging learning experiences because of their recent experience as learners themselves [3]. The emphasis on interactive teaching strategies is also consistent with broader principles of effective medical education [28].

Finally, participation appeared to influence future educational aspirations, with many trainees reporting increased confidence and enthusiasm for teaching careers. Given ongoing calls to expand the clinical educator workforce within primary care [19, 29], these findings suggest that structured near-peer teaching opportunities may play an important role in fostering future clinician-educators. The potential contribution to career satisfaction and portfolio career development may also have implications for retention and wellbeing within the GP workforce [30].

## Limitations and future research

While this study provides valuable insights into the experiences of GP trainees as near-peer educators, it has some limitations. The sample size was small and limited to a single teaching pilot scheme. The groups had self-selected to undertake the scheme, raising questions about transferability of the findings to the wider GP trainee cohort. Indeed, there may be great variability in competency and ability for GP trainees to take on this teaching role, which some GP trainers perceive as a barrier to near-peer teaching [14]. Furthermore, the findings are based on self-reported experiences and perceptions, which may be subject to recall bias and social desirability bias, with participants potentially emphasising the benefits of the programme or underreporting challenges. Future research could explore these themes across a larger and more diverse group of trainees, potentially including comparisons between different specialties or teaching contexts. However, the findings complement existing research highlighting the desire to teach amongst GPSTs, as well as the benefits of near-peer mentorship for both teacher and learner.

Additionally, longitudinal studies could investigate the long-term impact of such teaching experiences on trainees’ career trajectories and ongoing involvement in medical education.

## Implications for practice

The findings of this study have several implications for medical education and GP training:

1. *Incorporation of structured teaching opportunities*: GP training programs should consider incorporating formal teaching opportunities as part of their curriculum, given the significant benefits reported by trainees.
2. *Support and preparation*: Adequate support and preparation should be provided to doctors taking on teaching roles, particularly in areas such as time management and balancing clinical and teaching responsibilities. A training environment provides an ideal opportunity for this.
3. *Promoting medical education careers*: The positive experiences reported by trainees suggest that early exposure to teaching roles may help to cultivate future medical educators, addressing the ongoing need for skilled clinician-educators.

## Conclusion

This project demonstrates that participation in a near-peer teaching scheme can have significant positive impacts on GP trainees’ personal and professional development. Despite challenges, trainees reported numerous benefits, including improved clinical skills, enhanced job satisfaction, and the development of teaching and leadership abilities. As Speaker 7 states: “Yeah, I really loved it. I think I love the fact that we could do it now as trainees and not have to wait ‘til you CCT and become a GP because you get a lot of support now.” This enthusiasm for early involvement in teaching roles suggests that incorporating structured teaching opportunities into GP training programs may not only benefit the trainees themselves but also contribute to the development of a skilled and motivated future workforce of clinician-educators in general practice.

## Supplementary Information

Supplementary Material 1.

## Data Availability

The datasets used are not publicly available but are available on reasonable request.

## Abbreviations

CCT: Certification of Completion of Training
ECE: Early Clinical Experience
GP: General Practice
GPST: General Practice Specialist Trainee
GPSTEAM: General Practice Specialist Trainee Education and Mentoring
MRCGP: Membership of the Royal College of General Practitioners
RCGP: Royal College of General Practitioners
