# Supplementary material for: Evaluating GP trainees’ perceptions of teaching undergraduate medical students on clinical placements: a thematic analysis
Source: BMC Med Educ. 2026 Jul 1;26:1495. doi: 10.1186/s12909-026-09820-5 (PMC13591678; doi:10.1186/s12909-026-09820-5)
Supplement: Supplementary file 1 [file 12909_2026_9820_MOESM1_ESM.docx]

**GPST Questionnaire**

**Organisation**

I felt the PRIME training session prepared me adequately for clinical placement supervision

| Strongly disagree | Disagree | Neutral | Agree | Strongly Agree |
| --- | --- | --- | --- | --- |

I felt the medical school communicated clearly with me regarding the placement logistics

| Strongly disagree | Disagree | Neutral | Agree | Strongly Agree |
| --- | --- | --- | --- | --- |

I felt well supported by the medical school if I had any queries about placement supervision

| Strongly disagree | Disagree | Neutral | Agree | Strongly Agree |
| --- | --- | --- | --- | --- |

**Personal Development**

I felt being the main supervisor for students benefited me in the following ways:

Helped me with my own clinical learning

| Strongly disagree | Disagree | Neutral | Agree | Strongly Agree |
| --- | --- | --- | --- | --- |

Helped me fulfil my own GP training portfolio requirements

| Strongly disagree | Disagree | Neutral | Agree | Strongly Agree |
| --- | --- | --- | --- | --- |

Helped develop my teaching skills

| Strongly disagree | Disagree | Neutral | Agree | Strongly Agree |
| --- | --- | --- | --- | --- |

Helped develop other transferrable skills:

(i) Role modelling and leadership

| Strongly disagree | Disagree | Neutral | Agree | Strongly Agree |
| --- | --- | --- | --- | --- |

(ii) Time management

| Strongly disagree | Disagree | Neutral | Agree | Strongly Agree |
| --- | --- | --- | --- | --- |

(iii) Organisational skills

| Strongly disagree | Disagree | Neutral | Agree | Strongly Agree |
| --- | --- | --- | --- | --- |

(iv) Communication skills

| Strongly disagree | Disagree | Neutral | Agree | Strongly Agree |
| --- | --- | --- | --- | --- |

**Future plans**

As a result of this teaching pilot, I am more likely to want to develop my role in medical education in the future

| Strongly disagree | Disagree | Neutral | Agree | Strongly Agree |
| --- | --- | --- | --- | --- |

Ideally, I would like to actively pursue opportunities for more teaching/supervision:

| Within 2 years | 2-5 years | 5-10 years | 10+ years | Not interested in pursuing further |
| --- | --- | --- | --- | --- |

Please comment / explain your reasons further:

<Free Text>

**Final reflections**

Please comment on any particular highlights for you as a placement supervisor

<Free Text>

Please comment on any particular challenges you had or foresee facing as a placement supervisor

<Free Text>

Note: For the purpose of analysis 1 = Strongly disagree and 5 = Strongly agree
